# F161. BRAIN VOLUME CHANGE IN PATIENTS WITH SCHIZOPHRENIA AND ITS RELATION WITH ANTIPSYCHOTIC DRUG USE

**DOI:** 10.1093/schbul/sby017.692

**Published:** 2018-04-01

**Authors:** Fatma Kiras, Mustafa Yildiz, Yonca Anık

**Affiliations:** Kocaeli Üniversitesi

## Abstract

**Background:**

Schizophrenia is a chronic severe mental illness affecting 0.4% of the society with its early age of onset (generally between 15–25 years) which can be seen in people from all social strata, has recurrent episodes or relapses, and causes disability by deteriorating interpersonal and occupational functioning. Critical behavioral findings accelerated the efforts on understanding the neurobiology of this complex disorder. Although the nature of progressive structural brain change is not understood completely, identifying the role of antipsychotic drugs is important to comprehend how much of the progressive brain volume change is the result of schizophrenia.

The purpose of the study is to perform the volumetric brain magnetic resonance imaging (MRI) from patients with schizophrenia, to repeat the MRI after one year regular follow-up, to investigate whether brain volume-brain structures’ volume change by time and reveal its relationship with the type and dosage of antipsychotic drug (APD). This study is considered that would contribute to the discussions about the disease process of schizophrenia and the effect of APD use on structural changes in brain.

**Methods:**

Fifty-two patients diagnosed with schizophrenia according to DSM IV was participated to the study. Patients are followed for approximately 14 months. In the first interview, patients were applied to a sociodemographic form, The Structured Clinical Interview for DSM-IV (SCID-I), Positive and Negative Syndrome Scale (PANSS), Global Assessment of Functioning (GAF), Clinical Global Impression (CGI). After the first interviews, the patients who performed brain MR were arranged control meetings by the same doctor regularly (monthly, bimonthly, or when needed) until the second MR. In control meetings, patients were applied to clinical interview form, PANSS, GAF, and CGI. Drugs were recorded regularly by making sure that they took the drugs precisely with the help of their families. Chlorpromazine equivalent doses of APDs were calculated as milligram (mg) and turned into dose-year unit. Second MRIs were performed approximately after 14 months. In SPM program, paired sample t test was used to compare two MRIs and see whether gray matter volume was changed regardless the type of drugs the person used. Also, ANOVA was used to identify the relationship between the type of the drug taken between two MRIs and the volume change in gray matter by choosing the drug type as covariant. SPSS 22 was used to examine the relationship between the sociodemographic features of the patients and their brain volume findings.

**Results:**

From the patients participated in our study, 19 of them were female and 33 were male, mean age was 36.5, the level of education was 11 years, the mean onset of the disease was 24 and duration of the illness was 11 years. On the first MRI analyses, gray matter was negatively correlated with the age of the patients (r= -0,473, p<0,001) and the duration of the illness (r=-0,316, p<0,05), and positively correlated with the onset of the disease (r= 0,281, p<0,05) and the level of education (r= 0,321, p<0,05); while white matter positively correlated only with the level of education (r= 0,321, p<0,05). Throughout 14 months, no significant difference was found in brain volume change analysis with 95% confidence. Gray matter was not significantly correlated with APD dose, atypical antipsychotic drug (AAPD) use, and typical antipsychotic drug (TAPD) use.

**Discussion:**

It can be concluded that throughout 14 months period, brain volume of the patients with schizophrenia was not change significantly and the type and dose of APD used during this process did not have any effects on volume change.

